# Applying Reversible Mutations of Nodulation and Nitrogen-Fixation Genes to Study Social Cheating in *Rhizobium etli*-Legume Interaction

**DOI:** 10.1371/journal.pone.0070138

**Published:** 2013-07-26

**Authors:** Jun Ling, Huiming Zheng, David S. Katzianer, Hui Wang, Zengtao Zhong, Jun Zhu

**Affiliations:** 1 Department of Microbiology, Nanjing Agricultural University, Nanjing, China; 2 Department of Microbiology, Perelman School of Medicine, University of Pennsylvania, Philadelphia, Pennsylvania, United States of America; Portland State University, United States of America

## Abstract

Mutualisms are common in nature, though these symbioses can be quite permeable to cheaters in situations where one individual parasitizes the other by discontinuing cooperation yet still exploits the benefits of the partnership. In the *Rhizobium*-legume system, there are two separate contexts, namely nodulation and nitrogen fixation processes, by which resident *Rhizobium* individuals can benefit by cheating. Here, we constructed reversible and irreversible mutations in key nodulation and nitrogen-fixation pathways of *Rhizobium etli* and compared their interaction with plant hosts *Phaseolus vulgaris* to that of wild type. We show that *R. etli reversible* mutants deficient in nodulation factor production are capable of *intra-*specific cheating, wherein mutants exploit other *Rhizobium* individuals capable of producing these factors. Similarly, we show that *R. etli* mutants are also capable of cheating *inter-*specifically, colonizing the host legume yet contributing nothing to the partnership in terms of nitrogen fixation. Our findings indicate that cheating is possible in both of these frameworks, seemingly without damaging the stability of the mutualism itself. These results may potentially help explain observations suggesting that legume plants are commonly infected by multiple bacterial lineages during the nodulation process.

## Introduction

Rhizobia can form symbiosis nodules with legumes in which the bacteria fix atmospheric nitrogen into ammonia that can be utilized by the host plant. Two-way chemical signaling is required for this process. For example, *Rhizobium etli* is able to identify flavonoid substances released by its host legume *Phaseolus vulgaris* signaling it to produce nodulation factors (Nod), which in turn induce cortical cell division and form symbiosis nodules, where nitrogen is fixed [1]. Nodulation and nitrogen fixation is achieved by *nod* and *nif*/*fix* gene clusters, which play pivotal regulatory roles in symbiosis [2]. All Rhizobia strains contain the common nodulation genes *nodDABC*, which encode a transcription factor, as well as enzymes that are required to produce Nod factors [3]. In bacteroids that rhizobia differentiate in the infection thread, *nif* and *fix* genes are expressed through the FixL/FixJ two-component regulatory system and the transcriptional activator NifA [4] which leads to the production of nitrogenase and ultimately nitrogen fixation.

Due both to the ability of Rhizobia to form symbiotic nodules on host legumes and their ability to fix inert atmospheric nitrogen (N_2_) to plant-usable ammonia, mutualistic interactions are commonly studied within the Rhizobia-host system [5]. In this context, *Rhizobium* individuals cooperate with the plant host by fixing nitrogen into usable ammonia while benefiting from receiving host-derived resources, such as carbon [5]. Nitrogen-fixation, however, is an energetically costly process, and can be predicted to benefit Rhizobia only if the plant supplies efficient resources to outweigh the cost of ammonia production on the bacteria [6]. Given this cost, opportunity exists for non-cooperating partners to parasitize these social dynamics by reaping benefits (carbon or other resources) while failing to supply nitrogen-fixation in return [7]. It has been demonstrated that one way of mitigating this potential burden is through the differential allocation of host resources in attempts to punish these non-fixing “cheaters” within a community, though the mechanism of these sanctions is unknown [8]. Though these studies show that plants can reduce rhizobial nodule occupancy in scenarios where whole nodule cheating is forced, they do not address how plants respond to increased parasitism in the event of nodules containing a mixed population of both cooperating and non-cooperating strains, potential in nature given estimates of 12% to 32% of field soybean nodules containing double occupancy [9].

Similar dynamics can be studied within the colonization process itself. For effective colonization, Rhizobia secrete nodulation factors (Nod factors) which induce cortical cell division in their host legume [1]. For instance, previous studies have shown that it is possible for mutants deficient in nodulation to form mixed nodules with Nod factor-producing strains [10], suggesting that Rhizobia can hitch-hike within their own species during the nodule-formation process. This system, in turn, can be permeable to cheaters (nodulation deficient mutants) that benefit by colonizing with other individuals, allowing for host colonization and resource sequestration without the burden of producing Nod factors themselves. If a strong metabolic cost to produce these Nod factors exists, mutants deficient in nodulation can be expected to arise at high frequencies, as what typically happens with any common good [11]. As with nitrogen-fixation, the selective advantage gained by mutants deficient in nodulation factor production would depend upon the metabolic cost of production. In this study, we constructed reversible and irreversible mutations in the key *nod* and *nif* genes of *R. etli* and investigated their nodulation and N_2_-fixation behaviors when they were inoculated individually or co-inoculated with wild type strains. We show that *R. etli* is capable of both *intra-*specific cheating through the mutation of a key gene in the nodule-formation pathways as well as *inter-*specifically cheating their host through the mutation of a gene essential to nitrogen fixation, suggesting a mechanism for the existence of multiple bacterial strain populations in nature as well as a reason for their persistence.

## Materials and Methods

### Bacterial Strains, Vectors and Culture Conditions

*R. etli* CE3, a streptomycin-resistant derivative of *R. etli* CFN42 [12], was used in this study. *Escherichia coli* strains were grown on LB at 37°C [13]. *R. etli* strains were grown on peptone–yeast extract (PY) medium [12] or tryptone-yeast extract (TY) [14] at 28°C. All solid media contained 1.5% agar, and antibiotics were added at the following concentrations for *R. etli*: streptomycin (Sm), 100 µg/ml; kanamycin (Km), 100 µg/ml; tetracycline (Tc), 10 µg/ml; rifamycin (Rif), 5 µg/ml; chloramphenicol (Cm), 20 µg/ml. Sucrose was added to a final concentration of 10% in media for counter-selection of in-frame deletion strains and their derivatives [15]. Double-crossover deletions (irreversible) of *nodB* and *nifA* were constructed by cloning the regions flanking sequences of *nodB* and *nifA* with antibiotic resistant cassettes (chloramphenicol-resistant and tetracycline-resistant genes, respectively) into the suicide vector pEX18Gm containing a *sacB* counter-selectable marker [16]. The resulting plasmids were introduced into *R. etli* by conjugation and deletion mutants were selected for double homologous recombination events. Single-crossover mutations (reversible ) of *nodB* and *nifA* were constructed by cloning internal fragments of *nodB* and *nifA* into pVIK112 [17]. The resulting plasmids were then introduced into *R. etli* and insertional mutants were selected for single homologous recombination events. Under certain selective pressure, revertants through a second homologous recombination event of insertional mutants may persist.

### Nodulation Assays

*Phaseolus vulgaris* seeds were treated with 75% ethanol and 0.1% HgCl_2_ and were surface-sterilized. The treated seeds were then placed in Petri dishes and germinated in the dark at 28°C for 2–3 days. Seedlings were then immersed in approximately 10^8^ *R. etli* cells (either single inoculation or mixed inoculation) cultured in PY medium containing appropriate antibiotics and resuspended in sterilized water. After 20 mins, seedlings were transferred to pots containing sterile vermiculites and were grown in a plant growth chamber at 28°C with a 12 h/12 h day/night cycle. It has been shown that innocula containing high cell numbers may increase the frequency of mixed nodules [18]. The plants were watered as necessary with sterile nitrogen-free plant nutrient solution [19] for 30 days. The plants were then pulled out to count the number of nodules from both main roots and lateral roots.

To determine the nodule occupancy, nodules formed by mixed inoculation of wild type and mutants were surface-sterilized, crushed, and sequentially stabbed on PY agar containing selective antibiotics. After incubation at 28°C for 72 hrs, plates were inspected for the identity of strains. Nodule occupancy is based on at least 250 nodules isolated from 15–30 plants. To determine the competitive index of different mutant strains, colony forming units (CFUs) of bacterial cells in nodules containing mixed strains were determined by serial dilution and plating onto PY medium containing selective antibiotics. Competitive index (output ratio of two strains in a mixed nodule) was calculated as the output ratio of mutant to wild type. The data are a combination of four independent experiments. The following antibiotic-resistant patterns were used to distinguish each strain: wild type: Sm^R^, Rif^R^; *ΔnodB*: Sm^R^, Cm^R^ *; ΔnifA*: Sm^R^, Tc^R^ *; ::nodB* and *::nifA*: Sm^R^, Rif^S^, Km^R^; *nodB* and *nifA* revertants: Sm^R^, Rif^S^, Km^S^;

### Determination of Reversion Rates of Reversible Mutants

To determine the reversion rates of *::nodB* and *::nifA* grown outside of host plants, *::nodB* or ::*nifA* mutants were grown in PY medium in the absence of kanamycin at 28°C and were subcultured to fresh medium every other day. These mutants were also grown in the rhizosphere of *Phaseolus vulgaris* planted in autoclaved Vermiculite. Samples were withdrawn at the time points indicated and CFU of mutants (Sm^R^, Km^R^) and revertants (Sm^R^, Km^S^) was determined by plating onto PY medium plates containing streptomycin (Sm) only and streptomycin plus kanamycin (Km). The reversion rate was calculated by percentage of Km^S^ cells relative to total cell number. To determine the reversion rate in nodules, nodules were harvested 30-day post-inoculation, surface-sterilized, crushed, and resuspended in PY medium. CFU of mutants (Sm^R^, Km^R^), revertants (Sm^R^, Km^S^), and wild type (Sm^R^, Rif^R^) was determined by plating on PY medium plates containing Sm only, Sm+Rif, or Sm+Km. The reversion rate was calculated by percentage of Km^S^ revertants relative to total number of Sm^R^, Rif^S^ cells.

### Nitrogenase Activity Assays

Acetylene reduction assay was used to estimate nitrogenase activity following protocols previously described [20], [21]. Briefly, all nodules were collected from each plant, placed in 20 ml headspace bottles with 2 ml acetylene (10%), and incubated upside down at 28°C for 1 hr. Gas chromatography was conducted to measure peak height of ethylene and acetylene with 100 µl gas by an HP 6890 Series Gas Chromatograph System. The approximate nitrogenase activity is calculated as % of acetylene production per gram of nodule dry weight.

### Data Deposition

All raw data used in this study are available in Data S1.

## Results

### Construction of Irreversible and Reversible Mutants in Key Nodulation and N_2_ Fixation Genes

**Figure 1:**
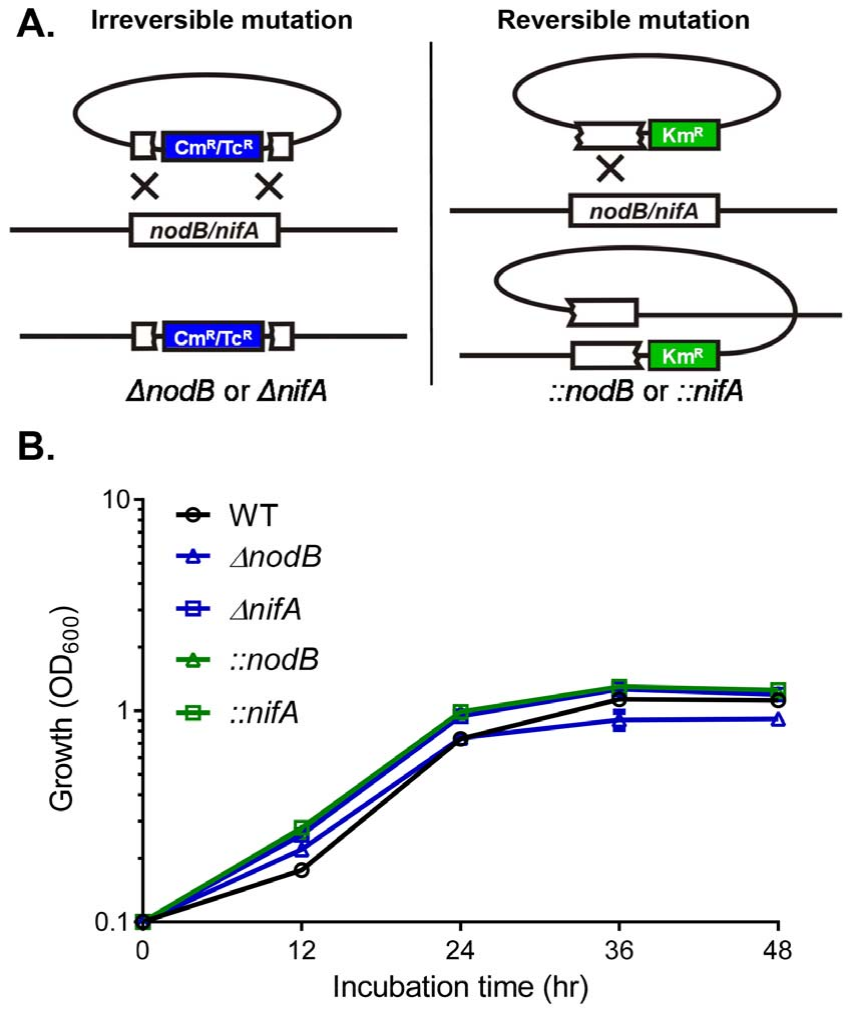
*R.* *etli nodB* and *nifA* mutation constructs. (**A**). The deletion constructs of *nodB* and *nifA* (left panel) were made by cloning flanking regions of *nodB* and *nifA* into a suicide vector harboring the *sacB* counter-selective marker. The resulting plasmids were then introduced into *R. etli* and double cross-over events were selected. The mutations generated by this method are permanent. The insertional mutants (right panel) were constructed by cloning internal fragments of *nodB* and *nifA* into a suicide vector. The resulting plasmids were then introduced into *R. etli* and single cross-over events were selected. The reversion to wild type may occur if the strains undergo a second cross-over event. Cm^R^: chloramphenicol-resistant gene; Tc^R^: tetracycline-resistant gene; Km^R^: kanamycin-resistant gene. (**B**). Growth of *R. etli* and its derivative mutants. The cultures were grown in PY medium at 28°C. At the time points indicated, OD_600_ was measured. Data are mean and s.d. of three independent experiments.

Rhizobium-legume symbiosis interaction has been studied extensively in recent years. However, how rhizobia interact with each other during the symbiosis and how the host affects this interaction are not fully understood. To study the *R. etli* cell-cell interaction in the context of the *R. etli-Phaseolus vulgaris* symbiosis, we constructed *R. etli* mutants in *nodB*, a common nodulation gene involved in the synthesis of Nod factors in rhizobia [22]; and in *nifA*, encoding a conserved transcriptional activator which is required for expressing N_2_-fixation related genes [23], [24]. We constructed two kinds of mutations: in addition to double-crossover replacement of *nodB* and *nifA* with antibiotic cassettes (annotated as *ΔnodB*, *ΔnifA*, respectively) (irreversible), we also made insertional mutants (reversible) by cloning the internal fragments of *nodB* and *nifA* into a suicide vector and introducing the resulting plasmids to select for the single homologous recombination events (annotated as *::nodB*, *::nifA*) (Fig. 1A). We reasoned that under certain selective pressure, the revertants (the insertional mutants may revert to wild type through a second homologous recombination event) may persist and we could detect such revertants by screening for the loss of the kanamycin-resistant cassettes. The mutants we constructed had no detectable effects on *in vitro* growth in the regular rich medium (Fig. 1B).

### Reversible and Irreversible *nodB* Mutants Present Distinct Nodulation Phenotypes

**Figure 2:**
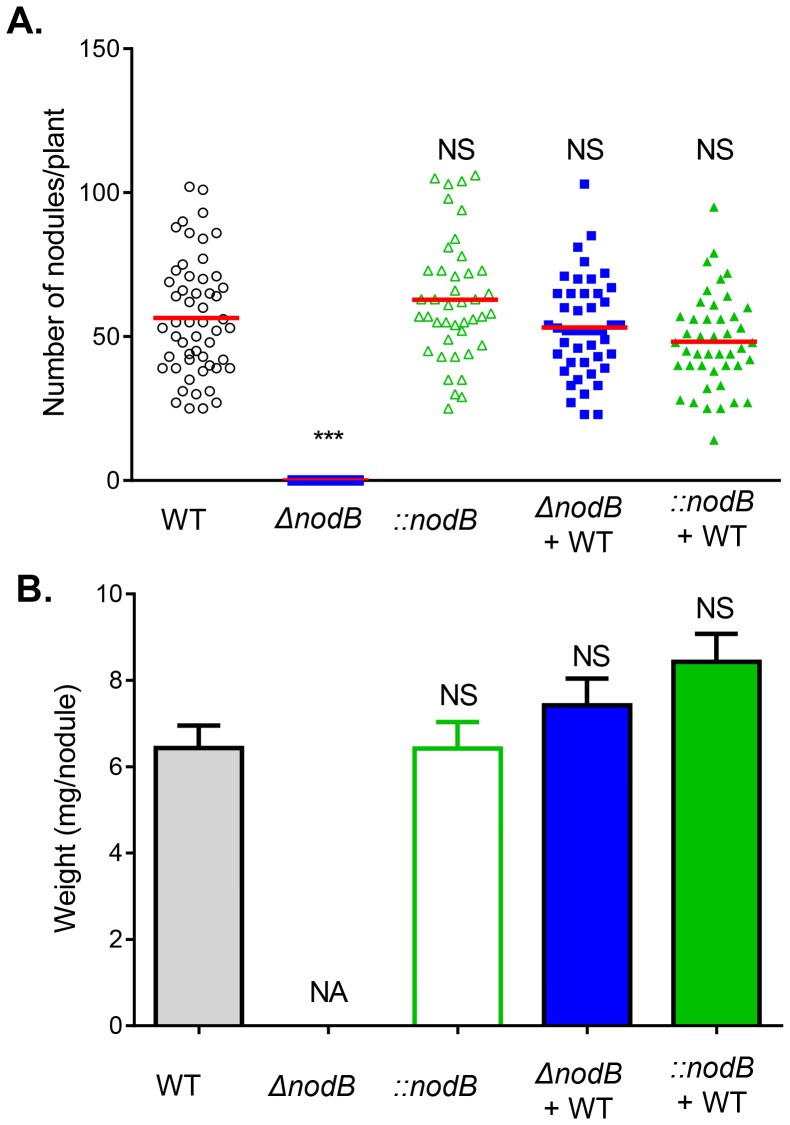
Nodule formation by wild type *R.* *etli* and its derivative *nodB* mutants. (**A**). The number of nodules on *Phaseolus vulgaris* formed 30 days post-inoculation by wild type (empty circles), *ΔnodB* (empty squares), *::nodB* (empty triangles), 1∶1 mixed inoculation of wild type and *ΔnodB* (filled squares), and mixed inoculation of wild type and *::nodB* (filled triangles). (**B**). Average biomass of the nodules. Data are the combination of six individual experiments. Statistical analysis was performed using the Student’s t-test comparing nodule formation to that of wild type. NS: no significance; ***: p<0.0001; NA: not applicable.

To examine the effect of *nodB* on nodulation, we immersed approximately 10^8^ cells of wild type, *ΔnodB*, *::nodB*, or a 1∶1 mixture of wild type with either *ΔnodB* or *::nodB*, with *Phaseolus vulgaris* seedlings. We examined the number of nodules on each plant after 30 days. Approximately 55 nodules/plant were formed when plants were inoculated with wild type (Fig. 2A, empty circles). As expected, no nodules were found on plants inoculated with *ΔnodB* mutants (empty squares), since NodB is essential for nodulation. Phenotypically, however,::*nodB* mutants did not display any nodulation defects, as the number of nodules formed by *::nodB* mutants was comparable to that of wild type (empty triangles). Interestingly, both mixed-inoculations (filled symbols) formed nodules similar in number to that of wild type. Additionally, the size of the nodules formed under different conditions was indistinguishable as the average weight of those nodules was similar to each other (Fig. 2B).

**Figure 3:**
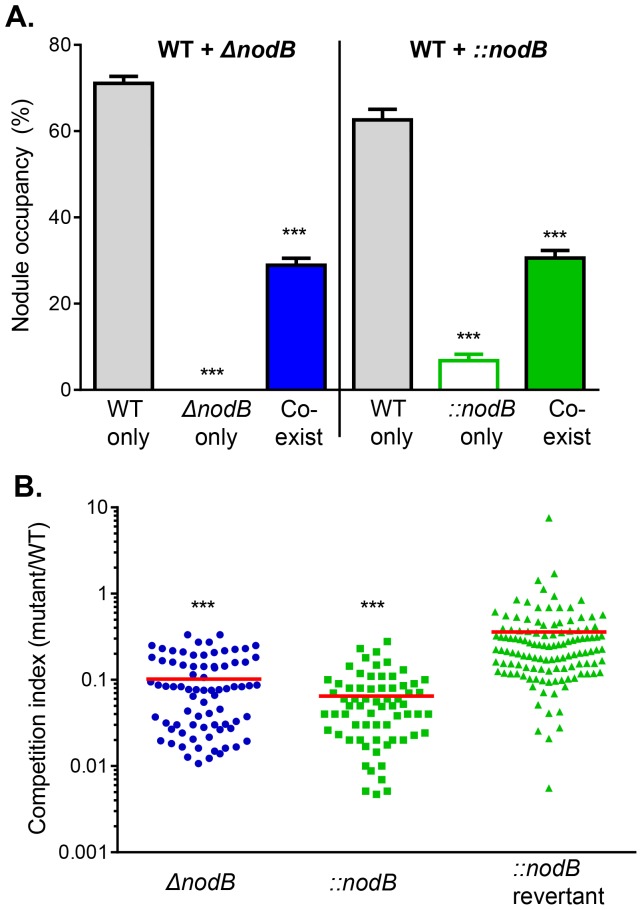
The *nodB* mutant competitiveness in nodulation. (**A**). Nodule occupancy. Nodules formed by 1∶1 mixed inoculation of wild type and Δ*nodB* (left panel) or ::*nodB* (right panel) were harvested 30-day post-inoculation, surface-sterilized, crushed, and sequentially stabbed on PY agar containing Sm+Rif, Sm+Cm, Sm+Km. After incubation at 28°C for 72 hrs, plates were inspected for the identity of strains. Nodule occupancy is based on 90 nodules isolated from 3 plants. Data are mean and s.d. of four independent experiments. Statistical analysis was performed using the Student’s t-test comparing to that of wild type. ***: p<0.0001. (**B**). Competitive index. For nodules containing mixed strains, CFU of wild type (Sm^R^, Rif^R^), *ΔnodB* (Sm^R^, Cm^R^), ::*nodB* (Sm^R^, Km^R^), *nodB* revertants (Sm^R^, Km^S^) was determined by plating onto PY medium plates containing Sm only, Sm+Rif, Sm+Cm, or Sm+Km. Competitive index was calculated as the output ratio of mutant to wild type. The data are combinations of four independent experiments. Statistical analysis was performed using the Student’s t-test comparing competitive index to that of *::nodB* revertant. ***: p<0.0001.

To analyze the competitiveness of *nodB* mutants, we examined the nodule occupancy in coinoculation experiments. From 300 nodules (total 78 plants) formed by mixed-inoculation of wild type and *ΔnodB* (Fig. 3A, left panel), none were occupied by *ΔnodB* alone, as expected. Interestingly, approximately 70% of the nodules were occupied by the wild type strain alone. The remaining 30% of nodules were a mixture of wild type and *ΔnodB* mutants. Among those nodules containing both strains, on average, the number of *ΔnodB* mutants was only 1/10 of the number of wild type in the same nodule, thus the competitive index was ≈ 0.1 (Fig. 3B, circles). When we examined nodule occupancy for nodules formed by mixed inoculation of wild type and *::nodB* mutants, we found that 6% nodules contained *::nodB* mutants alone, whereas 63% contained only the wild type strain, and 31% a wild type and *::nodB* mixture (Fig. 3A, right panel). Among the nodules containing both *::nodB* and wild type, the competitive index of *::nodB*(Sm^R^, Km^R^) was ≈ 0.06 (Fig. 3B, squares). These data suggest that *ΔnodB* mutants can only present in nodules with the help from wild type, whereas *::nodB* revertants can do so independent of wild type.

### Reversible *nodB* Mutants Displayed Selective Benefit during Nodulation

**Figure 4:**
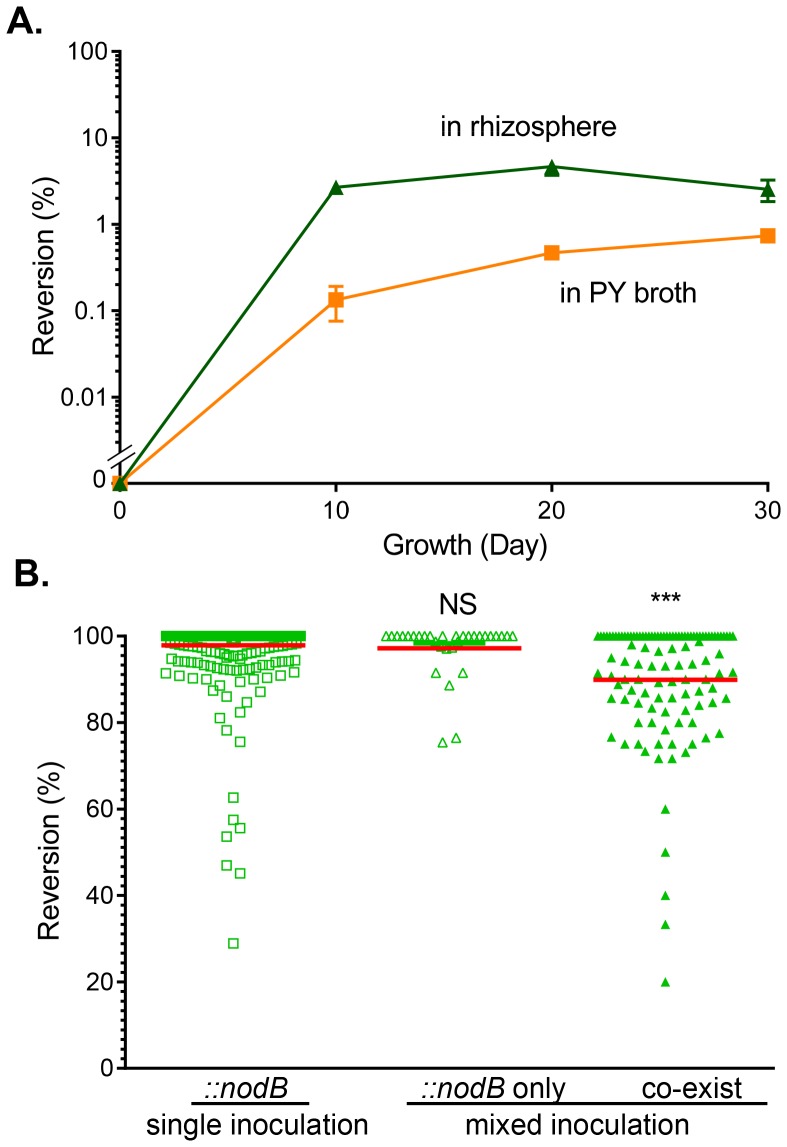
The *::nodB* reversion rate *in vitro* and *in planta*. (**A**). The reversion *in vitro*. *::nodB* mutants were grown in PY medium in the absence of kanamycin (squares) at 28°C and were subcultured to fresh medium every other day or grown in rhizosphere of *Phaseolus vulgaris* planted in autoclaved Vermiculite (triangles). Samples were withdrawn at the time points indicated and colony formation units (CFU) of *::nodB* (Sm^R^, Km^R^) and *nodB* revertants (Sm^R^, Km^S^) were determined by plating onto PY medium plates containing streptomycin (Sm) only and streptomycin plus kanamycin (Km). The reversion rate was calculated by the percentage of *nodB* revertants relative to total Sm^R^ cell number. Data are mean and s.d. of three independent experiments. (**B**). The reversion in nodules. Nodules formed by *::nodB* single inoculation (squares) or by*::nodB*-wild type mixed inoculation (triangles). Nodules formed by mixed inoculations contained either only *nodB* (empty triangles) or a mixture of *nodB* and wild type strains (filled triangles). Nodules were harvested 30-day post-inoculation, surface-sterilized, crushed, and resuspended in PY medium. Colony forming units (CFU) of ::*nodB* (Sm^R^, Km^R^), *nodB* revertants (Sm^R^, Km^S^), and wild type [Sm^R^, Rifampin (Rif^R^)] were determined by plating onto PY medium plates containing Sm only, Sm+Rif, or Sm+Km. The reversion rate was calculated by the percentage of *nodB* revertants (Km^S^) to total number (Sm^R^, Rif^S^) of cells. Statistical analyses were performed using the Student’s t-test comparing to reversion rate of *::nodB* mutants *ex planta.* NS: no significance; **: p<0.005.

We speculated that the reason the reversible *nodB* mutant (*::nodB*) could form nodules but not the deletion *nodB* mutant (*ΔnodB*) was because *::nodB* mutant can revert to wild type. To test this, we first examined the reversion rate of *::nodB* mutant *ex planta*. After 30-day growth of *::nodB* mutants in culture broth (squares) or around *Phaseolus vulgaris* roots (triangles) in the absence of antibiotics, less than 3% of *::nodB* mutants became kanamycin-sensitive (Fig. 4A). We confirmed by PCR analysis that those Km^S^ mutants were indeed revertants that contained the wild type copy of the *nodB* gene (data not shown). Next, we examined the reversion rate of *::nodB* mutants isolated from 30-day-old nodules. Strikingly, from 386 nodules formed by single-inoculation with *::nodB* mutants, 298 nodules contained 100% revertants, and on average, 97.9% of *::nodB* had reverted to wild type (Fig. 4B, squares). In the nodules formed by mixed inoculation of wild type and *::nodB* mutants, if nodules contained only *::nodB* mutants, 97.2% of *::nodB* mutants had reverted to wild type (Fig. 4B, empty triangles), explaining the data shown in Fig. 3A that “::*nodB* mutants” could exist alone in nodules (empty green bar). However, if nodules contained both wild type and *::nodB* mutants, the reversion rate was significantly reduced (Fig. 4B, filled triangles). Furthermore, the competitive index of the *::nodB* revertant was higher than those of *ΔnodB* and *::nodB* mutants (Fig. 3B, triangles). Taken together, these data suggest that due to the importance of NodB in nodulation, only those revertants are able to infect host and form nodules; however, while co-infected with wild type, the pressure for reversion is reduced. It is unclear whether the reversion happens prior to infection or during the infection.

### *nifA* Mutants have Advantages Over Wild Type in Nodules

**Figure 5:**
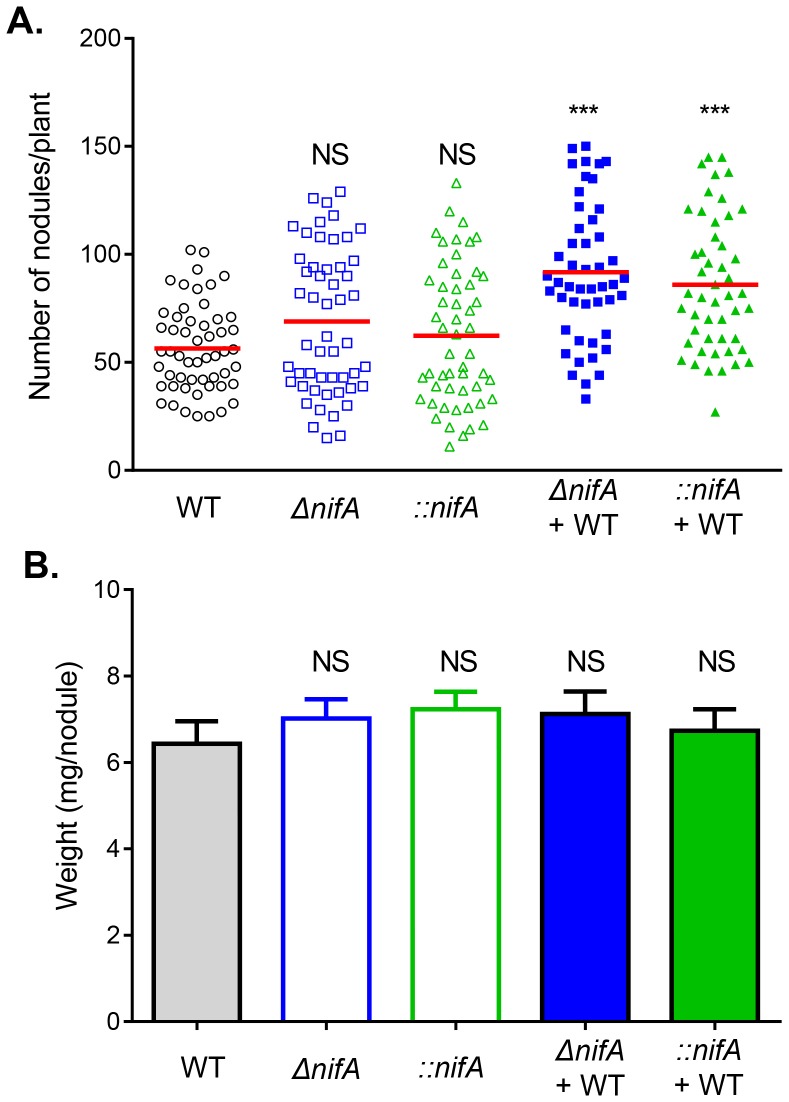
Nodule formation by wild type *R.* *etli* and its derivative *nifA* mutants. (**A**). The number of nodules on *Phaseolus vulgaris* formed 30 days post-inoculation by wild type (empty circles), *ΔnifA* (empty squares), *::nifA* (empty triangles), 1∶1 mixed inoculation of wild type and *ΔnifA* (filled squares), and mixed inoculation of wild type and *::nifA* (filled triangles). (**B**). Average biomass of the nodules. The data are the combination of 6 individual experiments. Statistical analyses were performed using the Student’s t-test acomparing to nodule formation in the wild type strain. NS: no significance; ***: p<0.0001.

In addition to studying *nodB*, a gene critical for nodulation, we also examined nodulation of *nifA* mutants (both deletion and insertion) with or without co-infecting with wild type. We then inoculated wild type, *ΔnifA*, *::nifA*, or 1∶1 mixture of wild type with either *ΔnifA* or *::nifA*, with *Phaseolus vulgaris* seedlings. We examined the number of nodules on each plant after 30 days. We found that single-inoculations of *ΔnifA* and *::nifA* both formed wild type levels of nodules (Fig. 5A, empty squares and triangles). Interestingly, mixed inoculations of wild type with either *ΔnifA* or *::nifA* induced plants to form statistically significantly more nodules than those of single inoculations (Fig. 5A, filled symbols). The size of nodules formed by different strains, however, was similar to each other (Fig. 5B). It is possible that *nifA* mutants save energy by not fixing nitrogen so that they have advantages for reproduction inside the nodules.

**Figure 6:**
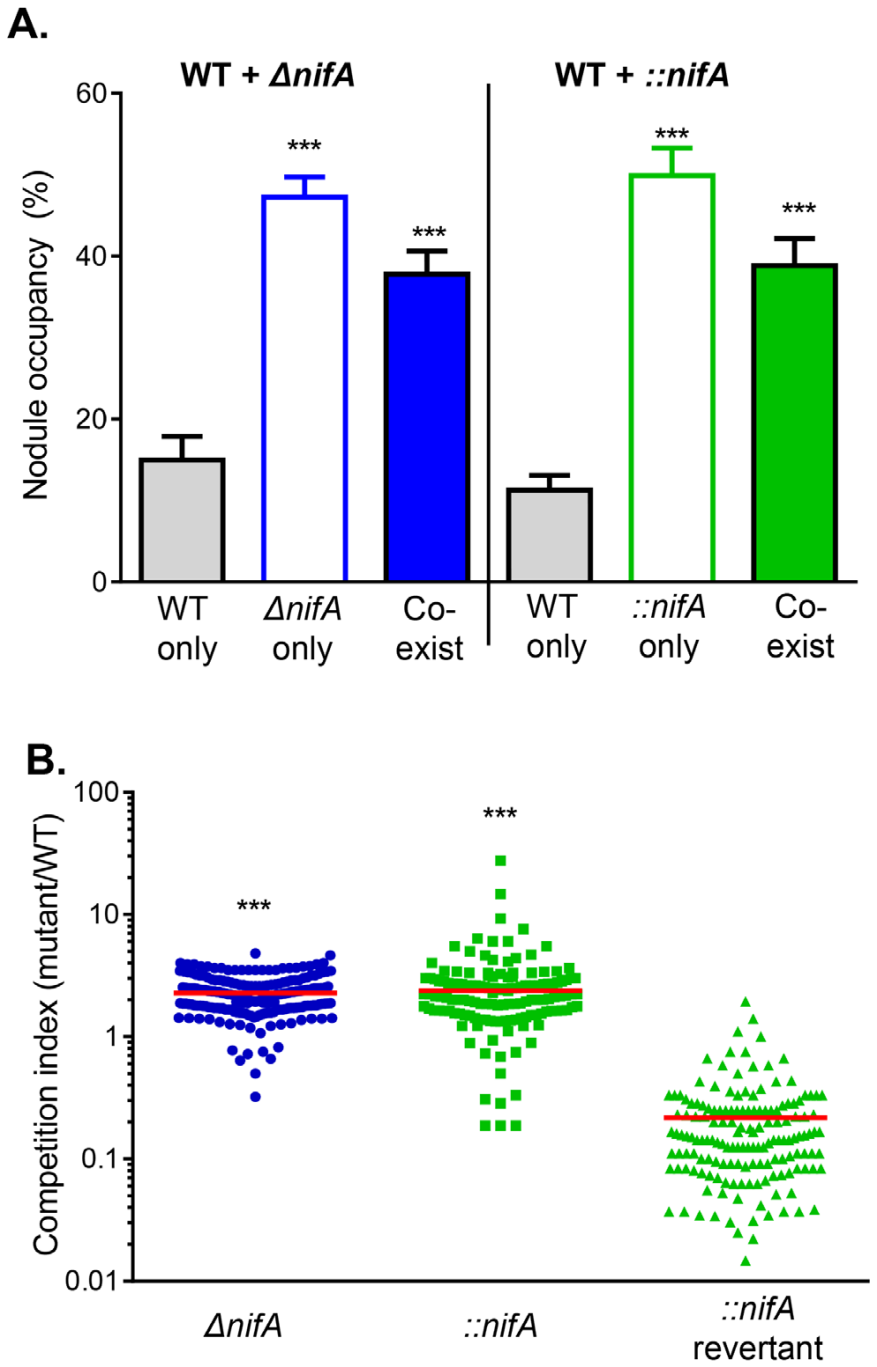
Competitiveness of *nifA* mutants in nodulation. (**A**). Nodule occupancy. Nodules formed by 1∶1 mixed inoculation of wild type and Δ*nifA* (left panel) or ::*nifA* (right panel) were harvested 30-day post-inoculation, surface-sterilized, crushed, and sequentially stabbed on PY agar containing Sm+Rif, Sm+Tc, Sm+Km. After incubation at 28°C for 72 hrs, plates were inspected for the identity of strains. Nodule occupancy is based on 90 nodules isolated from 3 plants. Data are mean and s.d. of four independent experiments. Statistical analyses were performed using the Student’s t-test comparing colonization to that of wild type. ***: p<0.0001. (**B**). Competitive index. For nodules containing mixed strains, CFU of wild type (Sm^R^, Rif^R^), *ΔnifA* (Sm^R^, Tc^R^), ::*nifA* (Sm^R^, Km^R^), *nifA*revertants (Sm^R^, Km^S^) was determined by plating on PY medium plates containing Sm only, Sm+Rif, Sm+Tc, or Sm+Km. Competitive index was calculated as the output ratio of mutant to wild type. The data are a combination of four independent experiments. Statistical analyses was performed using the Student’s t-test comparing competitive index to that of *::nifA revertant*. ***: p<0.0001.

We further analyzed nodule occupancy and competitiveness of mixed infections of wild type and *nifA* mutants. Both *ΔnifA* and *::nifA* mutants displayed nodulation advantages over wild type. The number of nodules containing *nifA* mutants (approximately 48% nodules containing only *nifA* mutants) was significantly higher than that of wild type alone (less than 15%) (Fig. 6A). For those nodules containing both wild type and mutant strains, *nifA* mutants were at least two-fold more than those of wild type and the competitive index of both *ΔnifA* and *::nifA* was over 2 (Fig. 6B, circles and squares). These data suggest that *nifA* reversible and irreversible mutant constructs behaved similarly in the nodulation process and mutations in N_2_ fixation genes may have advantages over wild type in nodulation.

### The Reversion Rate of Reversible *nifA* Mutants is Unchanged *in planta*

**Figure 7:**
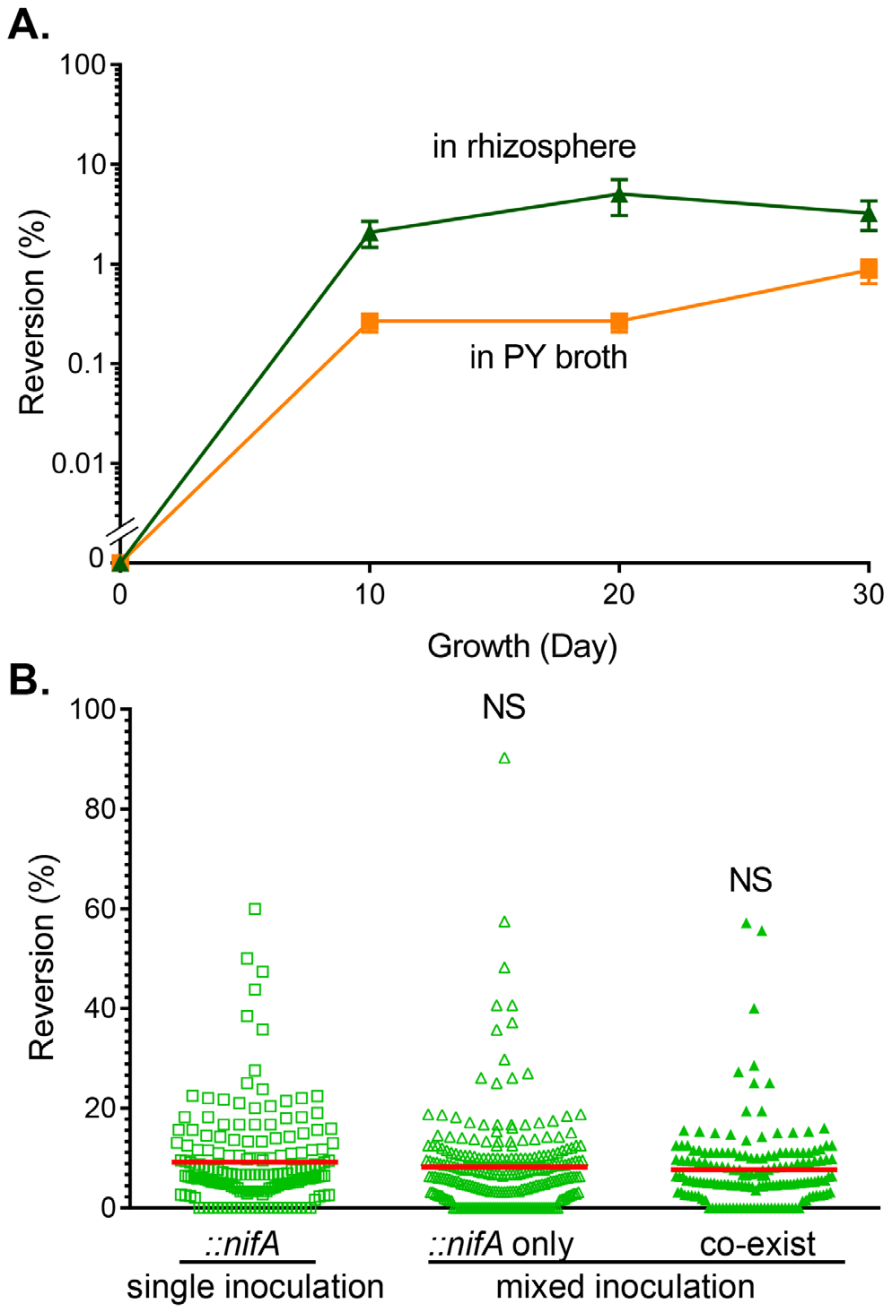
The *::nifA* reversion rate *in vitro* and *in planta*. (**A**). The reversion *in vitro*. *::nifA* strains were grown in PY medium in the absence of kanamycin (squares) at 28°C and were subcultured to fresh medium every other day or grown in rhizosphere of *Phaseolus vulgaris* planted in autoclaved Vermiculite (triangles). Samples were withdrawn at the time points indicated and colony forming units (CFU) of *::nifA* (Sm^R^, Km^R^) and *nifA* revertants (Sm^R^, Km^S^) were determined by plating on PY medium plates containing streptomycin (Sm) only and streptomycin plus kanamycin (Km). The reversion rate was calculated as percentage of *nifA* revertants (Km^S^) to total number of cells (Sm^R^). Data are the mean and s.d. of three independent experiments. (**B**). The reversion in nodules. Nodules formed by *::nifA* single inoculation (squares), or *::nifA*-wild type mixed inoculations (triangles). Of the mixed inoculations, nodules either contained only *nifA* (empty triangles) or a mixture of mutants with wild type strains (filled triangles). Nodules were harvested 30-day post-inoculation, surface-sterilized, crushed, and resuspended in PY medium. CFU of ::*nifA* (Sm^R^, Km^R^), *nifA* revertants (Sm^R^, Km^S^), and wild type (Sm^R^, Rif^R^) were determined by plating on PY medium plates containing Sm only, Sm+Rif, or Sm+Km. The reversion rate was calculated as percentage of *nifA* revertants (Km^S^) in total number of cells (Sm^R^, Rif^S^) cells. Statistical analyses were performed using the Student’s t-test comparing to reversion rate of ::*nifA* mutants *ex planta.* NS: no significance.

To further investigate the influence of the host on *nifA* mutant nodulation, we examined the reversion rate of *::nifA* to wild type under different growth conditions. The reversion rate of *::nifA* grown in the absence of antibiotic selection in rich medium (PY broth) for 30 days was less than 1%, whereas the reversion rate of *::nifA* grown around host roots was approximately 3% (Fig. 7A). Interestingly, the reversion rate of *::nifA* isolated from 30-day-old nodules was elevated to approximately 8% (Fig. 7B), though no difference was detected from nodules formed by single-inoculation of *::nifA* mutants or co-infection of wild type and *::nifA* mutants, suggesting that little selective pressure from the host was imposed on *nifA* mutants during this colonization duration.

### Co-infection of *nifA* Mutants with Wild Type does not Alter Nitrogen Fixation Rate

**Figure 8:**
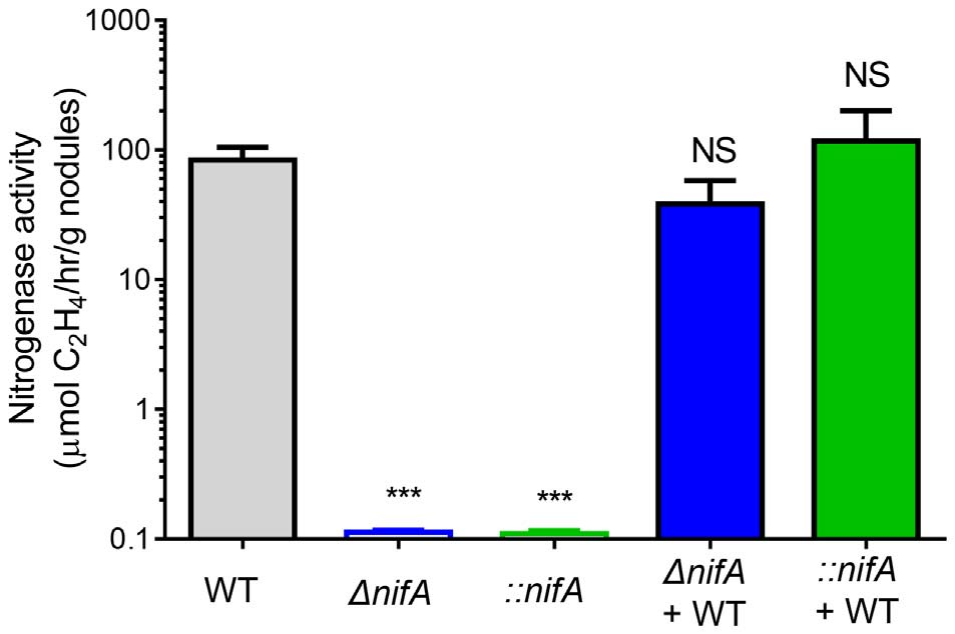
Nitrogenase activity in nodules. Nodules were formed by inoculation of a 1∶1 ratio of wild type to either Δ*nifA* or *::nifA* mutants. Nodules were harvested 30-days post-inoculation and acetylene reduction activity was determined using a HP 6890 Series gas chromatograph system (see Methods). Nitrogenase activity is expressed as % of acetylene production per gram of nodule dry weight. Data shows mean and s.d. of four independent experiments. Statistical analysis was performed using the Student’s t-test comparing nitrogenase activity to levels seen in nodules formed by a single inoculation of wild type. NS: no significance; ***: p<0.0001.

One prominent feature of rhizobium-legume interaction is that rhizobia are able to fix nitrogen after nodules are formed. To examine whether *nifA* mutants affect N_2_ fixation, we measured nitrogenase activity from 30-day-old nodules that were formed by different strains. Fig. 8 shows that, as expected, neither *ΔnifA*-induced nor *::nifA*-induced nodules had much nitrogenase activity, as NifA is essential for activating genes that are required for nitrogen fixation. However, the level of nitrogenase activity from the co-infection of *nifA* mutants with wild type was indistinguishable with nodules formed by wild type strains. These results are surprising as nodulation occupancy and competitive index of *nifA* mutants were higher than wild type (Fig. 6) and the *::nifA* reversion rate was less than 10% (Fig. 7). It is unclear whether, under such conditions, wild type rhizobia produce elevated nitrogenase activity to compensate for the loss resulted from *nifA* mutants or for some other unknown reason.

## Discussion

Social cheating can be common in microbial populations [25]–[28]. Cheating individuals, acting selfishly, can persist within a population despite being detrimental to the long-term survival of that population [29]. Similarly, “cheaters” can sometimes evade host pressure to cooperate in mutualistic relationships, particularly in rhizobium-legume interactions where mutualism efficiency, at least as defined in terms of nodule productivity, remains intact [5]. Here, we study induced cheating in the plant symbiont *Rhizobium etli* through two important processes thought necessary for success of the symbiosis: nodule formation and nitrogen fixation. Our results indicate that both *intra-* and *inter-*specific cheating is facilitated by cooperating strains in this system.

*Intra-*specific cheating, occurring when individuals within a population seemingly parasitize other individuals of the same species, has been shown to occur in a variety of natural systems [25]–[28]. In *Rhizobium*-host interactions, this cheating is predicted in situations such as nitrogen fixation to be thwarted by differential host sanctions imparted on non-cooperating nodules, likely due to allocating fewer resources to nodules supplying the host plant with little or no nitrogen [8]; however, few studies have examined the role of *intra-*specific cheating during the colonization process, a context potentially preceding the appropriation of resources and thus having little influence from the host. Here, we show that it is possible for mutants deficient in nodulation factor (Nod factors) production to colonize host nodules only if co-colonizing with Nod factor producing individuals, effectively “hitch-hiking” during the colonization process. We tested this using *nodB* mutants created by two different mechanisms (Fig. 1A) and theorized that those created by double-crossover deletions should not have the potential to revert to wild type during colonization, whereas those mutants created by single-crossover events could revert. In this context, deletion strains (*ΔnodB*) could not colonize host legumes by themselves, though mutants could co-exist in roughly 30% of nodules when co-inoculated with wild type strains (Fig. 3A), suggesting that host legumes can be primed for nodule production by strains producing Nod factors. The ability of *nod* deletion mutants to colonize only in the presence of wild type strains can imply somewhat of a redundancy to nodulation factor production in environments where wild type individuals are common.

Though deletion strains could not colonize by themselves, we investigated the colonization of *nodB* mutants created by single-crossover mutations (*::nodB*) because of the potential for these strains to revert to wild type through subsequent homologous recombination events. Nodule occupancy levels for experiments in which *::nodB* was co-inoculated with wild type strains were similar as those seen in the *ΔnodB*-wild type co-inoculations (Fig. 3A). Though nodule formation was possible when legumes were inoculated with only these mutants (*::nodB*) (Fig. 2A), 97.2% of *::nodB* mutants found in these nodules had reverted to wild type, which is predicted by the fact that nodulation factor production is necessary for the establishment of nodules on host legumes [3]. Taken together with the necessity for wild type co-colonization in *ΔnodB* mutants, it is likely that these reversion events in *::nodB* mutants are again simply necessary to establish nodule formation. As a whole, these data suggest that *nodB* mutants can potentially forego producing nodulation factors and instead rely on the production from neighboring individuals. Depending on the cost of nodulation factor production to wild type strains, these findings could have implications for a tragedy of the commons in which no individuals are able to colonize hosts due to a lack of nodulation factor production, though we have not surveyed isolates of *R. etli* to investigate whether *nod* mutants are common in natural systems.

Within the host nodule, it has been predicted that non-cooperating *Rhizobium* can be readily controlled, likely due to differential host sanctions meant to punish nodules deficient in nitrogen fixation [5]. Studies have shown that both Rhizobia occupancy per nodule and Rhizobia occupancy per nodule mass have decreased when forcing cheating by substituting an atmospheric N_2_:O_2_ mixture with Ar:O_2_ [8], though these experiments effectively force the entire rhizobia nodule population to cheat and may not represent host sanctions in the event of a mutant lineage deficient in nitrogen-fixation arising within an otherwise cooperating population. To test the effects of this, we created *nifA* mutants, again by two different mechanisms, hypothesizing that those mutants created by double-crossover deletions (*ΔnifA*) should not have the potential for reversion, while those created by single-crossover events (*::nifA*) could possibly revert to wild type. Surprisingly, single inoculation experiments with either *::nifA* or *ΔnifA* mutants produced nodule numbers and sizes similar to those seen in wild type controls, contrary to what is predicted by host sanctions (Fig. 5A). Similarly, single inoculation experiments showed that neither *::nifA* nor *ΔnifA* single inoculation experiments resulted in significantly different nodule weights compared to wild type controls (Fig. 5B). Although it is unclear why the host does not select against non-cooperating nodules, it is possible that host-derived sanctions could be detected after a longer growth period.

Though host sanctions are typically studied in contexts where whole nodules are forced to cheat, legume plants are typically infected with several bacterial lineages [5]. Within individual nodules, it could be expected that mutant bacterial lineages deficient in nitrogen-fixation could endure a selective advantage due to diminished metabolic burden, sweeping to high frequencies or fixation within the population. To study this, legumes were co-inoculated with either wild type strains and their *::nifA* reversible mutant derivatives or wild type and their *ΔnifA* deletion derivatives. Surprisingly, in both cases, co-inoculations formed more nodules per plant (Fig. 5A) compared to wild type and single-inoculations of each of the mutants, though the average weight of each nodule remained similar (Fig. 5B). Within the nodules, roughly 50% contained only the mutant strain and approximately 40% contained a mixed population of wild type and mutants (for both *::nifA* and *ΔnifA*). From these mixed populations, competitive index was measured and both mutants were found to have selective advantages relative to wild type strains, as expected by the decreased metabolic costs endured by lacking nitrogenase activity. Similarly, in *::nifA* reversible mutants, reversion rate was low both in single inoculations as well as in mixed inoculations, likely due to the costs associated with reverting. Interestingly, nitrogenase activity in co-inoculation experiments showed similar levels to those seen in wild type. While nodules inoculated with mutant strains by themselves could not reduce acetylene, the high levels of nitrogenase activity seen in mixed inoculations could suggest that wild type strains can increase fixation of atmospheric nitrogen, though we have not investigated expression levels of *nifA* in these instances. As host sanctions would likely be subjected to the nodule as a whole, this finding could imply that, in a mixed population of cooperating and non-cooperating strains, cooperating individuals could increase nitrogenase activity to account for the deficit due to cheaters, likely in attempts to avoid sanctions imparted on the nodule as a whole, thus punishing both cooperating and non-cooperating strains.

In this study, we show both that *Rhizobium* mutants can cheat during the nodule colonization process as well as suggest mechanisms for the stable persistence of these cheaters within the population. As it is likely that natural *Rhizobium*-plant mutualisms contain many distinct bacterial lineages, this study implies a potentially relevant manner in which to study this social parasitism in the natural context, as well as a reason for the persistence of many lineages within a population.

## Supporting Information

Data S1 (XLSX)
